# Human enteroendocrine cell responses to infection with *Chlamydia trachomatis*: a microarray study

**DOI:** 10.1186/1757-4749-6-24

**Published:** 2014-06-16

**Authors:** Aldona Dlugosz, Sandra Muschiol, Katherina Zakikhany, Ghazaleh Assadi, Mauro D’Amato, Greger Lindberg

**Affiliations:** 1 Karolinska Institutet, Department of Medicine, Division of Gastroenterology and Hepatology, Karolinska University Hospital, Gastrocentrum Huddinge K63, Stockholm, Sweden; 2 Karolinska Institutet, Department of Microbiology, Tumor and Cell Biology (MTC), Stockholm, Sweden; 3 Swedish Institute for Communicable Disease Control, Stockholm, Sweden; 4 Karolinska Institutet, Department of Biosciences and Nutrition, Stockholm, Sweden

**Keywords:** Enteroendocrine cells, *Chlamydia trachomatis*, Infection, Microarray

## Abstract

**Background:**

Enteroendocrine cells (EEC) are highly specialized cells producing signalling molecules vital to the normal functions of the gut. Recently, we showed altered protein distribution in *Chlamydia* infected EEC *in vitro.* The aim of this study was to perform a microarray analysis of the response pattern of EEC from both large and small bowel to infection *in vitro,* using *Chlamydia trachomatis* infection as a model.

**Methods:**

Two human EEC lines: LCC-18, derived from a neuroendocrine colonic tumour, and CNDT-2, derived from a small intestinal carcinoid, were infected using cultured *C. trachomatis* serovar LGV II strain 434 (ATCC VR-902B). Penicillin G was used to induce persistent infection. We used microarray analysis (Affymetrix GeneChip®) for studying changes in gene expression at different stages of infection.

**Results:**

Twenty-four hours after active and persistent infection, 66 and 411 genes in LCC-18 and 68 and 170 genes in CNDT-2 cells, respectively showed mean expression ratios >2-fold compared to non-infected cells. These genes encoded factors regulating apoptosis, cell differentiation, transcription regulation, cytokine activity, amine biosynthesis and vesicular transport. We found significant differences in gene transcription levels between persistently infected and non-infected cells in 10 genes coding for different solute carrier transporters (SLC) and in 5 genes related to endocrine function (*GABARAPL1*, *GRIP1*, *DRD2*, *SYT5* and *SYT7*).

**Conclusions:**

Infected EEC cells exhibit cell-type specific patterns related to vesicular transport, secretion and neurotransmitters. EEC play a pivotal role in regulation of gut motility and an impairment of enteroendocrine function can contribute to motility disorders.

## Background

Enteroendocrine cells (EEC) are present in the gastrointestinal (GI) tract (mucosa of the stomach, small intestine, colon, and rectum) and are highly specialized cells that produce hormones and other signalling substances that are vital to the normal function of the gut. Due to their diffuse localisation, EEC are difficult to study and so far, their role in the pathogenesis of bowel disorders has been only little explored. EEC play a pivotal role in sensory signalling for the enteric reflex regulating intestinal propulsive motility, secretion, and visceral hyperalgesia [1]. Alteration of endocrine cell function, particularly in the context of serotonin (5-HT), has been shown to be associated in a number of GI diseases including inflammatory bowel disease (IBD), coeliac disease and irritable bowel syndrome (IBS) [2-4]. The association between alterations in the production of gut hormones from EEC and various GI diseases emphasizes the significance of these cells in intestinal homeostasis.

EEC can be activated by bacterial ligands like lipopolysaccharide LPS [5] and may play a role in immune activation and in the regulation of gut inflammation. Altered endocrine function of the gut accompanies an inflammatory immune response [6]. Recently, our group found altered serotonin and chromogranin-A distribution in EEC infected with *Chlamydia trachomatis*[7]*.* In line with the microscopical findings, we found a significant down-regulation of the gene coding for the vesicular monoamine transporter (VMAT1) in infected compared with non-infected EEC (P < 0.05). Bacteria of the genus *Chlamydia* are gram-negative, obligate intracellular pathogens that can infect a wide range of eukaryotic cells and cause chronic and acute diseases in humans and animals. All *Chlamydia* species share a common biphasic developmental cycle in which the organism alternates between two developmental forms, the elementary body (EB), the infectious form of the organism and the reticulate body (RB), the metabolically active but non-infectious form of the bacterium. Upon attachment and internalization, EBs differentiate into RBs, that multiply inside a membrane-bound vacuole termed inclusion. RBs eventually differentiate back into EBs, which upon their exit from the host cell can start a new round of infection. In addition to such acute chlamydial infection, Chlamydia can persist within infected host cells, a condition that has been associated with a number of chronic diseases. Chlamydial persistence has been described as a viable but non-cultivable growth stage resulting in a long-term relationship with the infected host cell. Such relationships can be established in vitro through exposure to antibiotics [8].

The aim of this study was to evaluate the response pattern of enteroendocrine cells to intracellular bacteria *in vitro* using microarray technology. Based on previous results from our laboratory we used *C. trachomatis* as the infectious agent for studying gene expression alterations upon bacterial infection of EEC (2). Analysis of genome-wide expression patterns on the host during infection provides an insight into how the host recognizes and process a pathogen [9]. We assessed transcription profiles of EEC lines from both the large bowel and the small bowel after infection with *C. trachomatis*.

## Results

**Figure 1:**
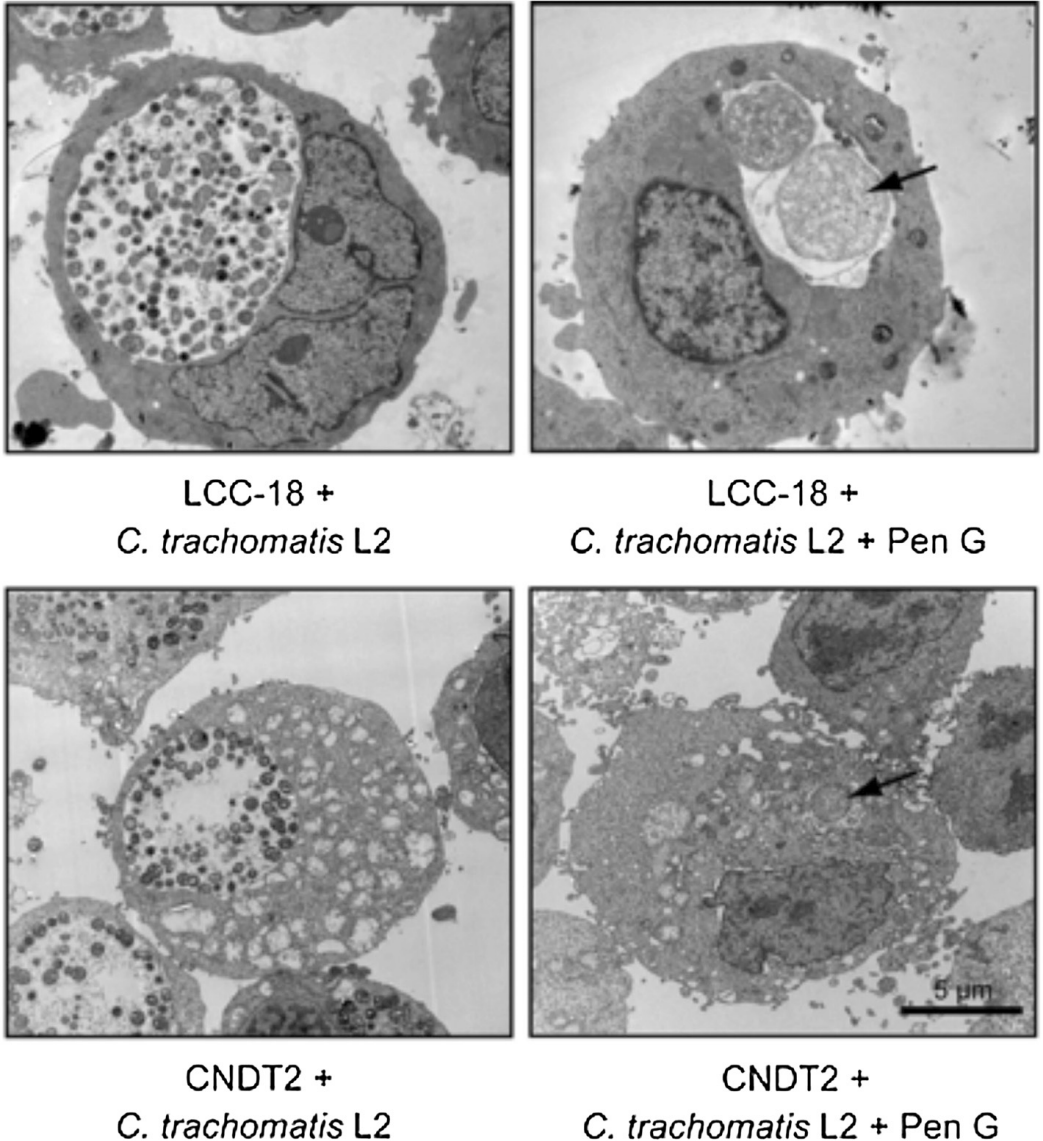
**LCC-18 and CNDT-2 cells actively and persistently infected with C*hlamydia trachomatis *L2.** Growth of *C. trachomatis,* manifested through inclusions containing both elementary bodies (EB) and reticulate bodies (RB) at 24 h post infection (p.i.), could be observed in both cell lines. In EEC treated with penicillin G enlarged, aberrant bacteria (arrows) reminiscent of persistent growth forms can be seen.

With TEM we confirmed active and persistent infection caused by *C. trachomatis* in enteroendocrine cells, originating from both large and small bowel. As shown in Figure 1, growth of *C. trachomatis,* manifested through inclusions containing both elementary bodies (EB) and reticulate bodies (RB) at 24 h post infection (p.i.), could be observed for both cell lines. When EECs were treated with penG we observed enlarged, aberrant bacteria reminiscent of persistent growth forms.

In order to screen host-cell gene regulation in response to *C. trachomatis* infection we used microarrays representing 30 000 human genes. We studied gene changes (up or down regulation) at the same time point (24 h post infection) in both active and persistent infection. The time point at 24 h was chosen as it represents a period of active metabolism and binary fission of *C. trachomatis* reticulate bodies within the host cell.

### Cell line specific responses to *C. trachomatis* active and persistent infection

We identified 66 differentially transcribed genes or expressed sequence tags (ESTs) (61 up-regulated) in LCC-18 cells with active *C.trachomatis* infection compared to non-infected cells and 411 (108 up-regulated) in persistently infected LCC-18 cells compared to non-infected cells. Twelve differentially transcribed genes (11 up-regulated) were identical for both active and persistent infection of LCC-18 cells. In order to identify different gene clusters among differentially transcribed genes we employed the DAVID Functional Annotation Tool. Based on this analysis four main clusters encoding genes involved in apoptosis GO:0006915, transcription regulation activity GO:0045449, vesicle mediated transport GO:0016192 and group of genes intrinsic to membrane GO:0031224 were identified.

**Figure 2:**
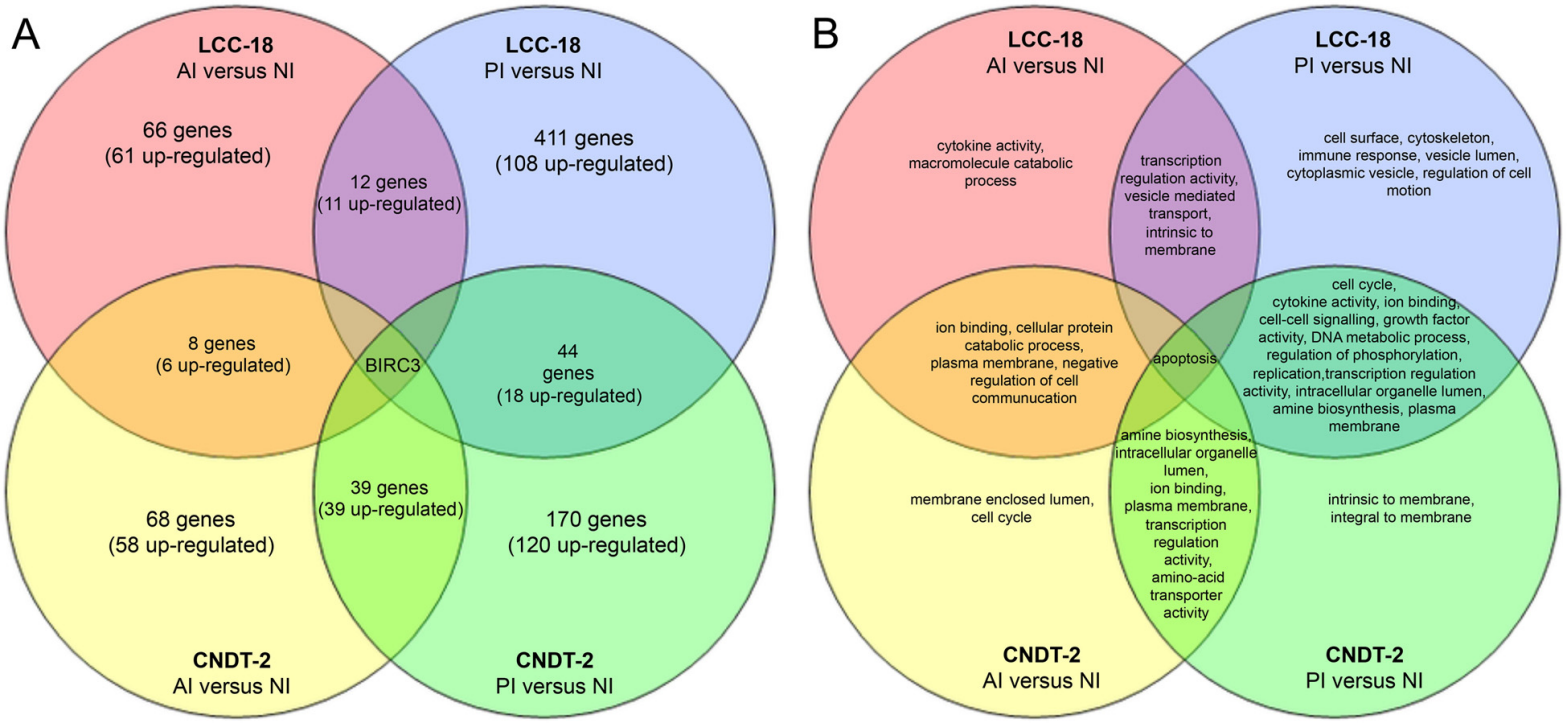
**Cell line specific responses and gene signatures of active and persistent*C. trachomatis *infection. A**. Differentially transcribed genes (up or down-regulated by >2-fold) in active (AI) or persistent (PI) infection of LCC-18 and CNDT-2 cells compared to non-infected cells (NI). We identified 66 differentially transcribed genes (61 up-regulated) in LCC-18 cells with active *C. trachomatis* infection compared to non-infected cells and 411 (108 up-regulated) in persistently infected LCC-18 cells compared to non-infected cells. Twelve differentially transcribed genes (11 up-regulated) were identical for both active and persistent infection of LCC-18 cells. In CNDT-2 cells, we identified 68 differentially transcribed genes when infected with *C. trachomatis* (58 up-regulated) and respectively 170 differentially transcribed genes or ESTs (120 up-regulated) in persistently infected CNDT-2 cells compared to non infected cells. Thirty-nine differentially transcribed genes (all up-regulated) were identical for both active and persistent infection of CNDT-2 cells. Baculoviral IAP repeat-containing 3 (*BIRC3*) was the only gene that was up-regulated in both cell lines during active and persistent infection. **B**. Schematic presentation of differentially transcribed genes clusters in active (AI) and persistent (PI) infection compared to non-infected cells (NI) in LCC-18 and CNDT-2 cells. Gene clustering based on Gene Ontology (GO) was performed using the Database for Annotation, Visualization and Integrated Discovery (DAVID) (http://david.abcc.ncifcrf.gov).

In CNDT-2 cells, we identified 68 differentially transcribed genes or expressed sequence tags (ESTs) when infected with *C. trachomatis* (58 up-regulated) and respectively 170 differentially transcribed genes or ESTs (120 up-regulated) in persistently infected CNDT-2 cells compared to non infected cells. Thirty-nine differentially transcribed genes (all up-regulated) were identical for both active and persistent infection of CNDT-2 cells. Genes were grouped in 8 clusters (amine biosynthesis GO:0009309, apoptosis GO:0006915, intracellular organelle lumen GO:0070013, plasma membrane GO:0005886, ion-binding GO:0043167, transcription regulator activity GO:0045449, intracellular non-membrane-bounded organelle GO:0043232, amino-acid transporter activity GO:0015171) (Figure 2).

Tables 1, 2, 3 and 4 show the most prominent differentially transcribed genes in the two cell lines. The presented genes are up or down-regulated by >3 fold in studied cell lines.

**Table 1:** **Differentially transcribed host genes up or down-regulated by >3-fold at 24 h after *C. trachomatis *** **infection of CNDT-2 cells compared to non-infected**

**Affymetrix ID**	**Gene name**	**Gene symbol**	**Log2 fold change**	**Absolute fold change**
**8102800**	Solute carrier family 7, (cationic amino acid transporter, y + system) member 11	SLC7A11	3,50	11,28
**8154381**	Chromosome 9 open reading frame 150	C9orf150	3,25	9,51
**7982868**	ChaC, cation transport regulator homolog 1 (E. coli)	CHAC1	2,63	6,18
**7928308**	DNA-damage-inducible transcript 4	DDIT4	2,41	5,33
**7902290**	Hystathionase (cystathionine gamma-lyase)	CTH	1,89	3,72
**8178086**	Heat shock 70 kDa protein 1A /// heat shock 70 kDa protein 1B	HSPA1A /// HSPA1B	-1,78	3,43
**8179324**				
**8118314**				
**8179322**				
**8118310**				
**8166585**	Hypothetical locus FLJ32742	FLJ32742	1,77	3,41
**7920873**	Small nucleolar RNA, H/ACA box 42	SNORA42	1,74	3,35
**7899436**	Sestrin 2	SESN2	1,66	3,17
**8139737**	Phosphoserine phosphatase	PSPH	1,66	3,17
**8060344**	Tribbles homolog 3 (Drosophila)	TRIB3	1,62	3,08
**8122724**	UL16 binding protein 1	ULBP1	1,61	3,06
**8143341**	Jumonji C domain containing histone demethylase 1 homolog D (S. cerevisiae)	JHDM1D	1,61	3,06
**8084219**	Kelch-like 24 (Drosophila)	KLHL24	1,59	3,03

Presented genes are involved in cellular amino-acid biosynthesis, stress response, carboxylic and organic acid biosynthetic process, amine biosynthetic process. Gene clustering based on Gene Ontology identified two clusters: apoptosis and ion binding.

**Table 2:** **Differentially transcribed host genes up or down-regulated by >3-fold at 24 h after *C. trachomatis *** **infection of LCC-18 cells compared to non-infected**

**Affymetrix ID**	**Gene name**	**Gene symbol**	**Log2 fold change**	**Absolute fold change**
**7943413**	Baculoviral IAP repeat-containing 3	BIRC3	2,14	4,41
**8124650**	Ubiquitin D /// gamma-aminobutyric acid (GABA) B receptor, 1	UBD /// GABBR1	2,00	4,00
**8178295**				
**7954090**	Epithelial membrane protein 1	EMP1	1,96	3,90
**8122265**	Tumor necrosis factor, alpha-induced protein 3	TNFAIP3	1,95	3,87
**7984259**	RNA, U5B small nuclear 1	RNU5B-1	1,72	3,29
**7908072**	Laminin, gamma 2	LAMC2	1,65	3,13

Gene clustering based on Gene Ontology identified two clusters: apoptosis and cellular protein catabolic process.

**Table 3:** **Differentially transcribed host genes up or down-regulated by >3-fold at 24 h after *C. trachomatis *** **persistent infection of CNDT-2 cells compared to non-infected**

**Affymetrix ID**	**Gene name**	**Gene symbol**	**Log2 fold change**	**Absolute fold change**
**8102800**	Solute carrier family 7, (cationic amino acid transporter, y + system) member 11	SLC7A11	3,77	13,56
**7982868**	ChaC, cation transport regulator homolog 1 (E. coli)	CHAC1	3,28	9,71
**7928308**	DNA-damage-inducible transcript 4	DDIT4	2,47	5,55
**8146934**	Lymphocyte antigen 96	LY96	2,09	4,26
**8154381**	Chromosome 9 open reading frame 150	C9orf150	1,96	3,89
**8143341**	Jumonji C domain containing histone demethylase 1 homolog D (S. cerevisiae)	JHDM1D	1,93	3,81
**8122724**	UL16 binding protein 1	ULBP1	1,85	3,61
**8174361**	TSC22 domain family, member 3	TSC22D3	1,75	3,36
**8122202**	v-myb myeloblastosis viral oncogene homolog (avian)	MYB	-1,73	3,31
**7912157**	ERBB receptor feedback inhibitor 1	ERRFI1	1,71	3,27
**8156043**	Phosphoserine aminotransferase 1	PSAT1	1,71	3,27
**8139737**	Phosphoserine phosphatase	PSPH	1,70	3,26
**7899436**	Sestrin 2	SESN2	1,65	3,14
**7902290**	Cystathionase (cystathionine gamma-lyase)	CTH	1,59	3,02
**8049542**	Leucine rich repeat (in FLII) interacting protein 1	LRRFIP1	1,59	3,00

Gene clustering based on Gene Ontology identified five clusters: amine biosynthesis, apoptosis, transcription regulator activity, intrinsic to membrane and ion binding.

**Table 4:** **Differentially transcribed host genes up or down-regulated by >3-fold at 24 h after *C. trachomatis *** **persistent infection of LCC-18 cells compared to non-infected**

**Affymetrix ID**	**Gene name**	**Gene symbol**	**Log2 fold change**	**Absolute fold change**
**8027002**	Growth differentiation factor 15	GDF15	3,32	10,01
**7958262**	t-complex 11 (mouse)-like 2	TCP11L2	2,96	7,79
**7954090**	Epithelial membrane protein 1	EMP1	2,59	6,03
**8158671**	Argininosuccinate synthetase 1	ASS1	2,10	4,29
**8152703**	F-box protein 32	FBXO32	2,09	4,25
**7982868**	ChaC, cation transport regulator homolog 1 (E. coli)	CHAC1	2,09	4,25
**8048319**	Villin 1	VIL1	-2,04	4,10
**8066431**	Adenosine deaminase	ADA	-2,00	3,99
**8008885**	microRNA 21	MIR21	1,99	3,96
**8034122**	SPC24, NDC80 kinetochore complex component, homolog (S. cerevisiae)	SPC24	-1,96	3,89
**8119898**	Vascular endothelial growth factor A	VEGFA	1,91	3,77
**8117429**	Histone cluster 1, H2bi	HIST1H2BI	-1,88	3,69
**7919591**	Family with sequence similarity 72, member D /// family with sequence similarity 72,	FAM72D / FAM72A / FAM72B / FAM72C	-1,84	3,60
**8122724**	UL16 binding protein 1	ULBP1	1,81	3,49
**8007071**	Cell division cycle 6 homolog (S. cerevisiae)	CDC6	-1,80	3,49
**8113073**	Arrestin domain containing 3	ARRDC3	1,79	3,45
**8068684**	Family with sequence similarity 3, member B	FAM3B	-1,76	3,40
**7989146**	Meiosis-specific nuclear structural 1	MNS1	-1,70	3,24
**8109712**	Hyaluronan-mediated motility receptor (RHAMM)	HMMR	-1,68	3,20
**8113278**	Lix1 homolog (chicken)	LIX1	-1,66	3,15
**7953943**	GABA(A) receptor-associated protein like 1	GABARAPL1	1,65	3,15
**8071212**	CDC45 cell division cycle 45-like (S. cerevisiae)	CDC45L	-1,65	3,14
**8009243**	Chromosome 17 open reading frame 60	C17orf60	1,65	3,13
**8062064**	Myosin, heavy chain 7B, cardiac muscle, beta	MYH7B	-1,64	3,11
**8021470**	Phorbol-12-myristate-13-acetate-induced protein 1	PMAIP1	1,62	3,08
**8095744**	Amphiregulin	AREG	1,62	3,08
**7989647**	KIAA0101 /// casein kinase 1, gamma 1	KIAA0101 /// CSNK1G1	-1,61	3,06
**7914878**	Arsenic transactivated protein 1	LOC100289612	-1,61	3,05
**8165663**	Glycerol-3-phosphate acyltransferase, mitochondrial	GPAM	-1,60	3,04
**8077731**	Fanconi anemia, complementation group D2	FANCD2	-1,59	3,03
**8151871**	Cyclin E2	CCNE2	-1,59	3,03
**8157524**	Toll-like receptor 4	TLR4	1,59	3,03
**8104234**	Thyroid hormone receptor interactor 13	TRIP13	-1,59	3,01

Gene clustering based on Gene Ontology identified ten clusters: cell cycle, cell surface, regulation of apoptosis, organelle lumen, cytoskeleton, immune response, regulation of phosphorylation, cytokine activity, intrinsic to membrane and ion binding.

### Gene signatures of active and persistent *C. trachomatis* infection

Host responses to infection in the two cell lines during active chlamydial infection were similar. Only 8 differentially transcribed genes (6 up-regulated) were the same for both CNDT-2 and LCC-18 cell lines. Differentially transcribed gene clusters comprised genes involved in apoptosis GO:000006915, negative regulation of cell communication GO:0010648, ion binding GO:0043167, cellular protein catabolic process GO:0005886, cell cycle GO:0007048, membrane enclosed lumen GO:0031974, cytokine activity GO:0005125, and vesicle mediated transport GO:0016192.In persistent infection we found 44 differentially transcribed genes (18 up-regulated and 26 down-regulated) in both CNDT-2 and LCC-18 cells. Among them were genes involved in apoptosis GO:0006915, cytokine activity GO:0005125, growth factor activity GO:0008083, cell cycle GO:0007049, DNA metabolic process GO:0006259, intracellular organelle lumen GO:0070013, amine biosynthesis GO:0009309, transcription regulation activity GO:0045449, cell-cell signalling GO:0007267, regulation of phosphorylation GO:0042325, ion binding GO:0043167, and plasma membrane GO:0005886 (Figure 2). The remaining differentially transcribed genes in persistent infection were involved in immune response GO:0006955, vesicle lumen GO:0031983, cytoplasmic vesicle GO:0031410, vesicle mediated transport GO:0016192, intrinsic to membrane GO:0031224 and regulation of cell motion GO:0051270 (Figure 2).

Baculoviral IAP repeat-containing 3 (*BIRC3*) was the only gene that was up-regulated in both cell lines during active and persistent infection.

### Selected genes of interest

Grouping of particular genes into specific functional classes is arbitrary to a certain degree, because some genes may be involved in several cellular functions and pathways. In order to study cell type-specific responses to infection, we analyzed the expression of genes associated with transport and endocrine function.

We found significant differences in the gene transcription levels between persistently infected and non-infected cells of 10 genes coding for different solute carrier transporters (SLC) (Table 5).

**Table 5:** **Differentially transcribed genes coding for solute carrier families (SLC), up or down-regulated at 24 h after *C. trachomatis *** **persistent infection of CNDT-2 and LCC-18 cells compared to non-infected**

**Affymetrix ID**	**Gene name**	**Gene symbol**	**Log2 fold change**	**Absolute fold change**	**Log2 fold change**	**Absolute fold change**
			**LCC-18**	**LCC-18**	**CNDT-2**	**CNDT-2**
			**PI v NI**	**PI v NI**	**PI v NI**	**PI v NI**
			**Microarray/ qPCR**	**Microrray/qPCR**	**Microarray/qPCR**	**Microarray/qPCR**
**8042310**	Solute carrier family 1 (glutamate/neutral amino acid transporter), member 4	SLC1A4	NC	NC	1,37	2,58
			0,94	1,9	1,12	2,2
**8068361**	Solute carrier family 5 (sodium/myo-inositol cotransporter), member 3	SLC5A3	1,2	2,3	NC	NC
			1,28	2,4	0,18	1,1
**8003298**	Solute carrier family 7 (cationic amino acid transporter, y + system), member 5	SLC7A5	NC	NC	1,26	2,4
			-0,6	1,4	0,48	1,4
**8102800**	Solute carrier family 7, (cationic amino acid transporter, y + system) member 11	SLC7A11	0,84	1,8	3,76	13,6
			1,63	3,1	1.55	2,9
**8017843**	Solute carrier family 16, member 6 (monocarboxylic acid transporter 7	SLC16A6	1,26	2,4	0,88	1,8
			0,68	1,6	1,67	3,2
**8010673**	Solute carrier family 25 (mitochondrial carrier; dicarboxylate transporter), member 10	SLC25A10	-0,79	1,7	-1,07	2,1
			-0,11	1,1	-1,49	2,5
**8012450**	Solute carrier family 25, member 35	SLC25A35	-0,96	2	-0,43	1,4
			-0,49	1,4	-0,79	1,7
**8162586**	Solute carrier family 35,member D2 UDP-N-acetylglucosamine/UDP-glucose/GDP-mannose transporter	SLC35D2	0,96	2	0,57	1,5
			0,74	1,7	0,8	1,7
**7962559**	Solute carrier family 38, member 4, Na(+)-coupled neutral amino acid transporter	SLC38A4	1,48	2,8	-1,1	2,2
			0,92	1,9	-0,56	1,4
**7965964**	Solute carrier family 41, member 2, plasma-membrane magnesium transporter	SLC41A2	0,4	1,3	1,18	2,3
			0,45	1,4	1,62	3,1

A fold change > 2 was observed in at least one comparison. Microarray results and validation of candidate genes using qPCR.

PI = persistent infection, NI = non-infected cells. Log2 fold change “-“ = down- regulated,“+” = up-regulated. NC = no change.

We also found significant differences in the expression of genes related to glutamate transport and synthesis: *SLC1A4*, *SLC7A11*, argininosuccinate synthetase 1 (*ASS1*) and glutamate receptor interacting protein 1 (*GRIP 1*) were up-regulated in persistently infected cells compared to non-infected cells. We identified 5 genes related to endocrine function that were differentially transcribed in persistent infection compared to non-infected cells but we found differences between studied cell lines. *GABARAPL1* and *SYT5* were up-regulated in both cell lines whereas *GRIP1*, *DRD2*, and *SYT7* were differentially transcribed only in LCC-18 cells (Table 6).

**Table 6:** **Differentially transcribed genes related to EEC function, up or down-regulated at 24 h after *C. trachomatis *** **persistent infection of CNDT-2 and LCC-18 cells compared to non-infected**

**Affymetrix ID**	**Gene name**	**Gene symbol**	**Log2 fold change**	**Absolute fold change**	**Log2 fold change**	**Absolute fold change**
			**LCC-18**	**LCC-18**	**CNDT-2**	**CNDT-2**
			**PI v NI**	**PI v NI**	**PI v NI**	**PI v NI**
**7953943**	GABA(A) receptor-associated protein like 1	GABARAPL	1,65	3,2	1,10	2,0
**7964759**	glutamate receptor interacting protein 1	GRIP1	1,14	2,2	NC	NC
**7951703**	Dopamine receptor 2	DRD2	1,02	2,0	NC	NC
**8039378**	Synaptotagmin5	SYT5	1,3	2,5	0,54	1,5
**7948588**	Synaptotagmin7	SYT7	-1,15	2,1	NC	NC

A fold change >2 was observed in at least one comparison. PI = persistent infection, NI = non- infected cells. Log2 fold change “-“ = down- regulated, “+” = up-regulated. NC = no change.

Although many similarities were found between the two cell lines we also found many differences in the response to infection. Some of these could be due to differences in basal gene transcription between the two studied cell lines. For example, basal expressions of receptors for gamma-aminobutyric acid: (GABA)A receptor, alpha 1 (*GABRA1*), (GABA)A receptor, alpha 3 (*GABRA3*), (GABA)A receptor, beta 2 (*GABRB2*) and (GABA)B receptor 1 (*GABBR1*) were significantly lower in CNDT-2 than in LCC-18, whereas (GABA)A receptor-associated protein like 1 (*GABARAPL1*) expression was lower in LCC-18 cells (data available at http://www.ncbi.nlm.nih.gov/geo/query/acc.cgi?acc=GSE58151).

### Confirmation using qPCR

The changes in expression of selected genes were validated by qPCR (Table 5). This process validated 10 genes coding for different solute carrier transporters (SLC1A4, SLC5A3, SLC7A5, SLC7A11, SLC16A6, SLC25A10, SLC25A35, SLC35D2, SLC38A4, SLC41A2). The changes in the expression levels of the validated genes between persistently infected and non-infected cells were paralleled in microarray and qPCR experiments in both cell lines.

## Discussion

Several studies have described *Chlamydia*-induced transcriptome changes in epithelial cells [10-12]. Our study is the first to describe such changes in enteroendocrine cells. Studying EEC response to infection seems to be very important since the adult human intestine is home to between 1000 and 1150 prevalent bacterial species [13]. The role of EEC in immune activation and promotion of inflammation in the gut has been postulated before [14-16]. A recent study showed that regenerating islet-derived gene REG4 coding for protein associated with epithelial inflammation was expressed in serotonin-producing enteroendocrine cells and expanded to epithelial cells of the upper colonic crypts during inflammation [17]. Alterations in the number of EEC have been observed in response to different bacterial, viral and parasitic infections of the GI tract [18,19] but the impact of a bacterial infection on EEC themselves has never been studied. Previous studies on *Chlamydia* infection of epithelial cells showed alterations in genes encoding apoptosis inhibitors, regulators of cell differentiation, components of the cytoskeleton, transcription factors, proinflammatory cytokines and cellular growth factors and cell cycle [18,20,21]. Our results showed that the impact of *Chlamydia* infection on apoptosis (*BIRC3, EMP1, CHAC1*), immune response, host cell structure and cell cycle in EEC is similar to what has been observed in epithelial cells [21,22].

The expression of *BIRC3* (also known as *cIAP2* or *HIAP*) [23], an apoptotic suppressor, was up-regulated in both cell lines during active and persistent infection. BIRC 3 is a component of the tumour necrosis factor receptor 2 (TNFR2) complex and, therefore, is a constituent of the TNF alpha (TNFα) signalling pathway [24]. BIRC3 has been demonstrated to inhibit cell death by directly repressing the proapoptotic activity of a family of cysteine proteases, caspases, as well as targeting proapoptotic components of the TNFα signalling pathway for ubiquitin degradation [25]. It was suggested that infection with Chlamydia protects mammalian host cells against apoptosis via BIRC3 up-regulation [26,27]. Inhibition of apoptosis and cytoskeleton modifications possibly accommodate chlamydial growth [9].

The ability of *Chlamydiae* to alter host cell proliferation and differentiation was manifested by up-regulation of growth factors: growth differentiation factor 15 (*GDF15*), vascular endothelial growth factor A (*VEGFA*), amphiregulin (*AREG*) and transforming growth factor beta 2 (*TGFB2*). *GDF15* was the most up-regulated gene in persistently infected LCC-18 cells (>10 fold change). GDF15 is a member of the TGFB family with a capacity to inhibit LPS-induced macrophage activation. GDF15 also provide significant neuroprotection with prominent effects on dopaminergic and serotonergic neurons [28].

The innate response upon *Chlamydia* infection of EEC is mediated by Toll-like receptor 4 (TLR4). *TLR4* was up-regulated in LCC-18 and lymphocyte antigen 96 (*LY96*) was up-regulated in CNDT-2. *LY96* encodes a protein, which associates with TLR 4 on the cell surface and confers responsiveness to LPS, thus providing a link between the receptor and LPS signalling.

Besides similarities in host-pathogen interaction between EEC and epithelial cells we found changes in gene expression in EEC that have never been described in epithelial cells. We found significant differences in gene transcription between persistently infected and non-infected cells in 10 genes coding for different solute carrier transporters (SLC) (Table 5). Transporters are the gatekeepers for all cells and organelles, controlling uptake and efflux of crucial compounds such as sugars, amino acids, nucleotides, inorganic ions and drugs. The SLC family include genes encoding passive transporters, ion coupled transporters and exchangers [29]. The biggest expression change was found for *SLC7A11* in CNDT-2 cells (>13-fold). SLC7A11 is responsible for a sodium-independent, high-affinity exchange of anionic amino acids with high specificity for anionic forms of cysteine and glutamate. Up-regulation of *SLC7A11* was previously reported in small bowel mucosa during cholera infection [30].

In addition to up-regulation of genes coding for two transporters involved in glutamate transport (*SLC7A11* and *SLC1A4*) we also found significant differences in the expression of other genes related to glutamate signalling (argininosuccinate synthetase 1 (*ASS1*) and glutamate receptor interacting protein 1(*GRIP 1*)) in persistently infected LCC-18 cells compared to non-infected cells. GABAA receptor-associated protein like 1 (*GABARAPL1*) was up-regulated during persistent infection in both cell lines. Glutamate and GABA (γ-aminobutyric acid) are the major neurotransmitters acting on specific G-protein-coupled receptors (the metabotropic glutamate (mGlu) and GABAB receptors) to modulate synaptic transport.

EEC provide an important link in transfer of information between gut and brain. Primary afferent neurons are excited indirectly by serotonine or hormones released from EEC. Sensory information from the gastrointestinal tract is transmitted via a glutamatergic synapse to neurons of the nucleus tractus solitarius, which integrate this sensory information to regulate autonomic functions and homeostasis. The integrated response is conveyed to the preganglionic neurons of the dorsal motor nucleus of the vagus using mainly GABA, glutamate and catecholamines as neurotransmitters [31]. Glutamate is involved in the regulation of gut motility and secretion by acting as an excitatory neurotransmitter in the gut [32]. Changes in glutamate related gene expression could be beneficial to *Chlamydia*. It was shown that glutamate could support chlamydial growth [33]. Ojcius et al. [34] demonstrated the stimulation of glutamate synthesis in cells infected with *Chlamydia psittaci*.

Genes associated with glutamate transport (*SLC7A11, SLC1A4*) were up-regulated in CNDT-2 cells and *ASS1*, which is involved in glutamate synthesis [35] and *GRIP1* were up-regulated in LCC-18 cells. GRIP1 is a synaptic protein interacting with glutamate receptors, playing an important role in their trafficking, synaptic targeting and recycling. GABARAP involves GRIP1 in the regulation of GABAA receptor function at inhibitory synapses [36].

GABA acts through a paracrine or autocrine mechanism via GABAA receptor activation [37]. This agent may play a similar role as a signalling molecule in endocrine organs and can for example inhibit the serotonin secretion [38]. The role of GABARAP binding proteins is not restricted to GABAA receptor transport but they participate in multiple biological processes, such as general vesicular transport and fusion events, autophagy and apoptosis. GABARAP appears to be associated with transport vesicles rather than the cell surface [39].

*DRD2* gene, encodes the D2 subtype of the dopamine receptor (another G-protein-coupled receptor), was up-regulated in persistent infection in LCC-18 cells. Dopamine (DA) is an enteric neurotransmitter and D2 seems to be an important mediator of neuronal responses to DA. Li et al. [40] showed that propulsive motility is increased in the gut of mice that lack D2 receptors suggesting that D2 is a major mediator of the effects of endogenous DA in the enteric nervous system (ENS).

Synaptotagmins *SYT5* and *SYT7* are other endocrine related genes that were differentially transcribed in persistent infection of LCC-18. Neurotransmitters, neuropeptides and hormones are released through the regulated exocytosis of SVs (synaptic vesicles) and LDCVs (large dense-core vesicles), a process that is controlled by calcium [41]. Ca^2+^ triggers many forms of exocytosis in different types of eukaryotic cells, for example synaptic vesicle exocytosis in neurons, granule exocytosis in mast cells, and hormone exocytosis in endocrine cells [42]. Synaptotagmins are a family of type-1-membrane proteins that share a common domain structure. Most synaptotagmins are located in brain and endocrine cells, and some of these synaptotagmins bind to phospholipids and calcium at levels that trigger regulated exocytosis of SVs and LDCVs [41]. Synaptotagmin 5 is a neuroendocrine cell-specific synaptotagmin responsible for phospholipid binding. Synaptotagmin 7 is a major Ca^2+^-sensor in endocrine LDCV exocytosis [42].

Our study provides new insights into the molecular events taking place in *Chlamydia* infection of EEC. We found that Chlamydia infected EEC may share some gene expression patterns with infected epithelial cells but they also seem to have some cell type-specific patterns related to vesicular transport and secretion, and neurotransmitters. We also observed differences in gene expression changes caused by *Chlamydia* infection between our two cell lines. It is well known that there are important differences in the luminal environments between the small and large intestines and we cannot exclude the influence of cell origins on obtained results. We found many differences in basal gene transcriptions (non-infected cells) between the two cell lines but infected cells seem to share the same genetic pattern independent of their origin.

Given the functions of EEC, it can be anticipated that altered function, in the presence of disease, might underpin symptom development. EEC clearly have multiple important functions in the control of appetite, GI motility and mucosal immunity. At present, EEC dysfunction related to various GI diseases is acknowledged, but remains poorly understood [43].

We do not have evidence that *Chlamydia* is the causative agent for motility disorders but we have shown that such an infection can influence EEC function. We studied the response pattern of enteroendocrine cells to intracellular bacteria *in vitro* using *C. trachomatis* as the infectious agent. Our findings can serve as a model of bacterial impact on EEC function but we cannot determine if our findings are *Chlamydia* specific or represent a general response to intracellular infection. Infection of mouse STC-1 enteroendocrine cells with *Salmonella typhimurium* was recently reported [44]. We successfully infected LCC18 with *Salmonella typhimurium* obtaining intracellular growth of these bacteria in EEC, but we have not analysed the effects of this infection on gene expression.

## Conclusions

Based on our findings that infected EEC exhibit cell-type specific gene expression patterns related to vesicular transport, secretion and neurotransmitter regulation our study sheds some light on the potential impact of bacteria on the exocrine function of EEC. Since EEC play a pivotal role in the regulation of gut motility any impairment of enteroendocrine function could potentially contribute to motility disorders.

## Methods

### Cell lines

We used two human enteroendocrine cell lines: LCC-18, derived from a neuroendocrine colonic tumour (a kind gift from K. Öberg, Uppsala University Hospital, Uppsala, Sweden); and CNDT-2, derived from a small intestinal carcinoid (a kind gift from Lee M. Ellis, Dept. of Surgical Oncology, The University of Texas, Houston, TX, USA). LCC-18 cells were maintained in RPMI 1640 (Invitrogen, Life Technologies) supplemented with 5% foetal bovine serum (FBS) (Hyclone, Thermo Scientific) 0.04 mg/ml transferrin (Sigma-Aldrich), 1.09 ng/ml b-estradiol (Sigma-Aldrich), 100U/ml insulin (please add supplier), 2.6 ng/ml hydrocortisone (Sigma-Aldrich), 30nM sodium selenite (Sigma-Aldrich,) and 25 mM HEPES (Invitrogen, Life Technologies). CNDT-2 cells were cultured in Advanced DMEM/F12 medium (Gibco, Life Technologies ) supplemented with 10% FBS, 1x MEM vitamin solution (Gibco, Life Technologies), 2 mM L-glutamine (Hyclone, Thermo Scientific) and 25 mM HEPES (Invitrogen, Life Technologies) Both cell lines were grown at 37°C in a 5% CO_2_ humidified environment. [7] LCC-18 are non-adherent and CNDT-2 cells grow adherent.

### Chlamydial strains and infection conditions

*Chlamydia trachomatis* L2 strain 434 (ATCC) was propagated in HeLa cells as previously described [45]. For infection, LCC-18 and CNDT-2 cells were inoculated with *Chlamydia* at a multiplicity of infection (MOI) of 0.5-1 for 1 h at 37°C. Monolayers of infected CNDT-2 cells were washed with PBS (Phosphate buffered saline) and incubated in fresh media media containing 8 μg/ml gentamycin (Invitrogen, Life Technologies) for 24 h. Infected LCC-18 cells were spun at 500 x *g* for 5 min to pellet the cells. The inoculum was removed and fresh media containing 8 μg/ml gentamycin (Invitrogen) to kill off extracellular bacteria was added. Infected LCC-18 cells were then incubated for 24 h. In order to induce persistent infection, LCC-18 and CNDT-2 cells were infected as described above and then incubated in medium containing 100 U/ml penicillin G (penG) (Sigma-Aldrich) for 24 h at 37°C.

### Transmission electron microscopy (TEM)

Cells were grown in 6-well plates and infected with *Chlamydia trachomatis* L2 in presence or absence of penicillin G as described. At 24 h post infection the cells were harvested, fixed in 0.1 M sodium cacodylate buffer (pH 7.4) containing 2% glutaraldehyde, 0.5% paraformaldehyde, 0.1 M sucrose and 3 mM CaCl_2_ and prepared for transmission electron microscopy as previously described [46] and analysed in a BioTWIN (Fei, The Netherlands). Digital images were obtained using a Veleta digital camera (Soft Imaging System, Germany).

### RNA extraction and microarray expression analysis

Total RNA was isolated from 1 x 10^6^ LCC-18 and CNDT-2 cells infected with *Chlamydia trachomatis* L2 (MOI 1) in presence or absence of penicillin G for 24 h. RNA samples were prepared using the RNeasy Mini kit (Qiagen) including an on-column DNase treatment step according to the manufacturer´s instructions. RNA concentrations were measured with a ND-1000 spectrophotometer (NanoDrop Technologies, USA) and RNA quality was assessed using the Agilent 2100 Bioanalyzer (Agilent Technologies Inc, USA). 250 ng of total RNA from each sample were used to generate amplified and biotinylated cDNA according to the Ambion WT Expression Kit (P/N 4425209 Rev B 05/2009) and Affymetrix GeneChip® WT Terminal Labeling and Hybridization User Manual (P/N 702808 Rev. 1, Affymetrix Inc., USA). GeneChip® ST Arrays (GeneChip® Human Gene 1.0 ST Array) were hybridized for 16 hours in a 45°C incubator, rotated at 60 rpm. According to the GeneChip® Expression Wash, Stain and Scan Manual (PN 702731 Rev 2, Affymetrix Inc., USA) the arrays were then washed and stained using the Fluidics Station 450 and finally scanned using the GeneChip® Scanner 3000 7G.

### Microarray data analysis

Analysis of the gene expression data was carried out using the freely available statistical computing software R (http://www.r-project.org) using packages available from the Bioconductor project (http://www.bioconductor.org). Raw data was normalized using the robust multi-array average (RMA) method first suggested by Li and Wong in 2001 [47,48]. In order to search for the differentially expressed genes between acute and persistent infection and the control samples (non-infected) an empirical Bayes moderated t-test [49] was applied using the ‘limma’ package [50]. The p-values were adjusted for multiple comparisons using the method of Benjamini and Hochberg [20]. Genes with an expression ratio >2-fold and adjusted p < 0.05 were regarded as differentially transcribed genes [21]. Gene clustering based on Gene Ontology (GO) was performed using the Database for Annotation, Visualization and Integrated Discovery (DAVID) (http://david.abcc.ncifcrf.gov).

The data discussed in this publication have been deposited in NCBI's Gene Expression Omnibus [51] and are accessible through GEO Series accession number GSE58151 (http://www.ncbi.nlm.nih.gov/geo/query/acc.cgi?acc=GSE58151).

### Confirmation of microarray results by use of RT-PCR

To verify the microarray results, qPCR was carried out on 10 genes coding for different solute carrier transporters that were up- or down-regulated at 24 h after *C. trachomatis* persistent infection of CNDT-2 and LCC-18 cells compared to non-infected.

Total RNA was isolated from 1 × 10^6^ LCC-18 and CNDT-2 cells (persistently infected at 24 h after infection and non-infected cells, as prepared for the microarray experiment) by using the RNeasy Mini kit (Qiagen) according to the manufacturer´s instructions. RNA concentrations were measured with a ND-1000 spectrophotometer (NanoDrop Technologies, USA), and 1,5 μg of total RNA was used for cDNA synthesis, performed with the SuperScript First-Strand Synthesis System (Invitrogen). Real-Time quantitative PCR (qPCR) was carried out using TaqMan Gene Expression Assays (Applied Biosystems) on 10 genes of interest (SLC1A4; Hs00161719_m1, SLC5A3; Hs00272857_s1, SLC7A5; Hs00185826_m1, SLC7A11; Hs00921938_m1, SLC16A6; Hs00190779_m1, SLC25A10; Hs00201730_m1, SLC25A35; Hs01390367_m1, SLC35D2; Hs00294687_m1, SLC38A4; Hs00394339_m1, SLC41A2; Hs00259767_m1). PCRs were performed in triplicate for each gene on the same sample, and expression values normalized for the endogenous control GAPDH. Gene expression was calculated with the comparative ΔΔCt method for relative quantification, and expressed as fold changes (arbitrary units) relative to an arbitrarily chosen reference sample.

## Competing interests

The authors have no competing interests to disclose.

## Authors’ contributions

AD designed the study, analyzed the data and wrote the paper, SM conducted cell culture, infection experiment and DNA extraction and wrote a paper, KZ performed DNA extraction and wrote the paper, GA performed the RT-PCR validation, MD analyzed the data and wrote the paper, GL designed the research study and wrote the paper. All authors read and approved the final manuscript.
